# From Grass to Protein: Assessing the Economic Viability of Mechanochemical-Assisted Extraction for Sustainable Food Production

**DOI:** 10.1021/acssuschemeng.5c12483

**Published:** 2026-01-23

**Authors:** Bernardo Castro-Dominguez, Oscar Selway Mindrina, Madhurima Dutta, Yubin Ding, Karl Behrendt, Anne Wambui Mumbi, Richard Green, Hannah S. Leese, Christopher J. Chuck

**Affiliations:** † Department of Chemical Engineering, 1555 University of Bath, Bath BA2 7AY, U.K.; ‡ Harper Adams Business School, 6000 Harper Adams University, Newport TF10 8NB, U.K.; § Department of Engineering, Harper Adams University, Newport TF10 8NB, U.K.; ∥ Anglia Ruskin University Writtle Faculty of Science & Engineering ARU, Lordship Road, Writtle, Chelmsford, CM1 3RR, U.K.

**Keywords:** grass biorefinery, mechanochemistry, protein extraction, techno-economic assessment, sustainable food systems

## Abstract

Grasslands represent one of the world’s largest yet most underexploited renewable biomass resources. Here, we present a techno-economic framework for transforming grass silage into edible protein and microbial lipids through mechanochemical and biocatalytic processing. Two biorefinery configurations were evaluated using stochastic and spatial modeling: a baseline system producing protein and biogas (Scenario 1) and an integrated design incorporating lipid fermentation (Scenario 2). Both achieve strong economic performance at industrial scale, with median net present values (NPVs) of £528 million and £1.21 billion, respectively, and protein production costs of £2.97–3.40 kg^–1^—comparable to plant-derived alternatives. Sensitivity analysis reveals that protein extraction efficiency and product price dominate profitability, while scale and coproduct valorisation drive the largest gains in expected NPV. Spatial simulations show that sourcing 33,333 t y^–1^ of wet silage (25% DM) is logistically feasible across UK grasslands at delivered costs of £51–58 t^–1^, enabling decentralised, regionally integrated deployment. Together, these results establish grass-based biorefineries as a scalable and economically credible route to sustainable protein production, bridging agricultural residues and food technology. The study provides quantitative guidance on how process yield, market development, and spatial logistics can be co-optimized to accelerate the emergence of a circular, pasture-driven bioeconomy.

## Graphical abstract

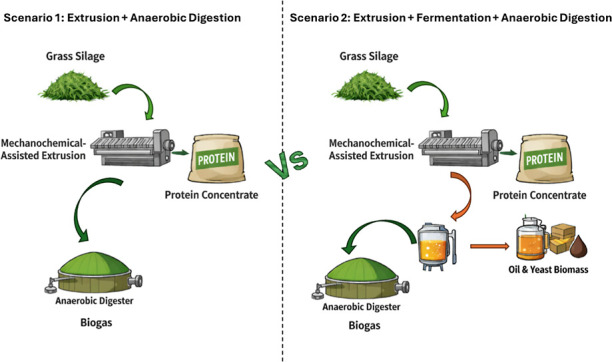

## 1 Introduction

Global protein supply for human consumption is under increasing pressure to meet the needs of a growing and more sustainability-conscious population. Traditional animal-based protein systems are responsible for over 60% of agriculture-related greenhouse gas emissions, require extensive land and water resources, and often depend on potentially inefficient and complex supply chains.^1^ In contrast, locally sourced plant proteins offer a promising alternative—provided that scalable, cost-effective, and nutritionally adequate extraction technologies can be developed.^2^

The United Kingdom presents a unique opportunity to pioneer such approaches. With over 12 million hectares dedicated to grassland (around 70% of the nation’s agricultural land^3^), the UK’s largest and most consistent biomass resource remains largely underutilized for direct human nutrition. Estimates suggest that more than 20 million tonnes of grass are harvestable annually from non-upland terrain, with a crude protein content of 17–32% of dry matter (DM) in fresh grass, representing a latent protein reservoir equivalent to nearly double the UK’s current dietary protein consumption.^4^ Yet humans cannot digest lignocellulose, the structural backbone of grass, necessitating technological intervention to liberate edible fractions. Silage was selected as the feedstock for this study rather than fresh grass due to its practical advantages for decentralised processing, including year-round availability, reduced seasonal variability, improved storability, and compatibility with existing agricultural infrastructure.

Mechanochemical-assisted extraction (MAE) has emerged as a leading candidate for sustainable biomass valorisation. Unlike conventional alkaline extraction with sodium hydroxide, which is effective but produces caustic waste, MAE employs mild bases such as sodium carbonate in conjunction with physical disruption (e.g., via twin-screw extrusion) to solubilize proteins while maintaining structural integrity and minimizing environmental harm. Twin-screw extrusion, in particular, offers a continuous, scalable, and reagent-efficient processing route.^5,6^

In recent laboratory-scale studies, we have demonstrated the potential of MAE in extracting functional proteins from various grasses. For example, extrusion of Moor grass transferred approximately 22% of the biomass into the liquid phase following mechanochemical disruption and pressing. The extracted protein showed a complete essential amino acid profile comparable to soy, and coextracted water-soluble vitamins—particularly B1, B2, B3, and B6—further enhanced its nutritional value. Although the functional properties (e.g., emulsifying and foaming capacity) of silage-derived protein were modest relative to egg or dairy proteins, its nutritional completeness and processing scalability make it a compelling ingredient for bulk applications^7^_._

The remaining biomass, rich in structural carbohydrates, can serve as feedstock for microbial fermentation, producing additional food-grade products such as mycoprotein and omega-rich lipids from oleaginous yeasts like *Metschnikowia pulcherrima*.^8^ Alternatively, the residual solid stream can be routed to anaerobic digestion (AD), where it is converted into biogas, primarily methane, for use as a renewable energy source. This dual-pathway flexibility enhances system resilience and resource efficiency, allowing for dynamic allocation based on market demand or infrastructural constraints. Together, these routes position grass silage as a cornerstone feedstock for decentralised, circular biomanufacturing platforms that support both food and energy security. Although protein recovery from green biomass has been demonstrated using a range of mechanical, enzymatic, and alkaline-assisted approaches, most prior studies focus on laboratory-scale performance metrics.^5,6^ Fewer studies integrate process economics, coproduct valorisation, and feedstock logistics, particularly for mechanochemical routes.^16^ This work addresses that gap through a combined techno-economic and spatial assessment.

This study focuses on evaluating the commercial feasibility of an MAE-based process for producing grass-derived protein powder from ensiled biomass. A detailed techno-economic assessment is presented, incorporating process design, capital and operating cost estimation. Given parameter uncertainty, Monte Carlo simulation techniques are used to quantify financial risk and performance drivers.^9^ Our goal is to bridge the gap between lab-scale innovation and industrial implementation, offering a rigorous evaluation of MAE as a foundation for future grass-based protein supply chains. This work establishes the first integrated mechanochemical–biocatalytic TEA for grass-based protein and lipid coproduction.

## 2 Methodology

### 2.1 Experimental Basis and Data Sources

All process parameters, including extraction efficiencies, product compositions, and material yields, were derived from experimental results obtained through mechanochemical-assisted extraction and downstream processing trials. These data provided the foundation for mass and energy balances in both process scenarios. Silage was produced using conventional agricultural ensiling practices prior to laboratory processing, ensuring representative moisture content and biomass stability for downstream experimentation. The experimental biomass consisted of grass-dominant silage, representative of mixed pasture systems. While botanical composition influences protein content and extractability, the techno-economic framework developed here is yield-driven rather than species-specific. Variability in species composition is therefore explored implicitly through sensitivity analysis on protein recovery and product yields. Detailed results supporting these values are presented in the Supporting Information in Section SI-1. Where experimental data were unavailable, standard literature correlations and validated process assumptions were applied to ensure consistency and reproducibility.

### 2.2 Process Configurations and Design Basis

Mechanochemical-assisted extraction (MAE) was implemented using a continuous twin-screw extrusion and pressing configuration, with sodium carbonate employed as a mild alkaline additive and no additional solvents. To assess the feasibility of valorising grass silage through MAE, two process scenarios were modeled that differ in how residual biomass is handled downstream of protein extraction. Both scenarios share a common front-end design for protein recovery but diverge in the pathways for managing the lignocellulosic residue. As shown in Table 1, both scenarios are designed for a silage processing capacity of 33,333 t y^–1^, aligned with pilot and medium-scale plant-based protein manufacturing.^10^ Based on an average crude protein content of 15 wt % in dry silage and assuming a theoretical maximum protein recovery of 15 g protein/100 g silage to model maximum potential output, the base case target is a protein powder output of 5000 t y^–1^. The detailed compositional analysis of the ensiled biomass used in this study, including dry matter content, total extractives, cellulose, hemicellulose, lignin, and protein content, is provided in Table SI1. It is important to clarify that the compositional values correspond to dried silage samples analyzed under controlled laboratory conditions, where the dry matter (DM) content is 95%. This value is significantly higher than what is typically observed in on-farm ensiling practices (which generally range between 30–40% DM) and was selected to enable accurate mass-normalized biochemical characterization of the biomass components. Consequently, the protein content (11.45 wt % on a dry basis) reflects the specific batch of silage used for lab-scale compositional analysis, rather than a representative or generalized field value. For the purposes of techno-economic modeling, a theoretical maximum protein content of 15 wt % was adopted to estimate best-case protein recovery potential, in line with values reported in the literature for high-quality forage and extractable protein fractions. This value is used as a starting assumption for process modeling and is later varied in the sensitivity analysis to reflect uncertainty and potential variability in feedstock quality and process yield.

**Table 1:** Design Basis and Expected Product Yields for the Silage Biorefinery^a^

feature	value	rationale
silage processing capacity	33,333 t y^–1^	experimental data: 15 g of protein/100 g of silage
protein production rate	5,000 t y^–1^	common medium-scale plant-based protein facilities.
oil rich yeast production rate	3,333 t y^–1^	experimental data: ∼10 g of yeast lipid/100 g of silage
lignin production rate	4,000 t y^–1^	experimental data: 12 g/100 g of silage
dry olid biomass for AD	28,500 t y^–1^	from mass balance (Section, S2)

a Values reflect annual output for a facility processing 33,333 tonnes of silage, based on experimental yield data for protein, lipids, and lignin recovery.

The selected capacity of 33,333 t y^–1^ reflects a balance between process intensification and realistic deployment in a decentralised manufacturing context. This scale is consistent with medium-sized facilities for plant-based protein production and is suitable for regional grassland catchments across the UK or Northern Europe. At this scale, the facility can operate continuously with high asset utilization, while remaining adaptable to existing agricultural and biowaste infrastructure.

#### 2.2.1 Scenario 1—Protein Production + Anaerobic Digestion

In this baseline scenario, the process is optimized solely for protein powder production (Figure 1a). After MAE, solubilized proteins are separated via hydrocyclone and concentrated using membrane filtration. Under optimized extrusion conditions—28 °C, 82 rpm, and a solid-to-liquid ratio of 1:31—experimental trials showed that approximately 22 wt % of the biomass was solubilized, with 52% of the total protein recovered in the liquid phase. The extracted protein fraction, recovered at 60 wt % purity (dry basis) following membrane concentration, exhibited a complete essential amino acid profile comparable to soy, as shown in Supporting Information Figure 1, with notably high glutamic acid content. Although not a purified isolate, the concentrate demonstrates nutritional adequacy for bulk plant-based formulations, particularly those targeting savory or umami-rich applications (Supporting Information Figure S1). The remaining solid fraction, comprising lignin-rich and carbohydrate-rich residues, is sent to AD. This converts the residual organics into biogas, primarily methane, which can be used on-site to offset thermal and electrical energy demand or exported to the grid. AD was selected due to its maturity, flexibility in handling variable lignocellulosic residues, and compatibility with decentralised energy systems. Digestate from the AD unit can be valorised as fertilizer or soil conditioner. Note that AD is treated as an external, mature valorisation route; indicative biogas yields and composition are provided for context, but reactor sizing and operation are not explicitly modeled.

**Figure 1:**
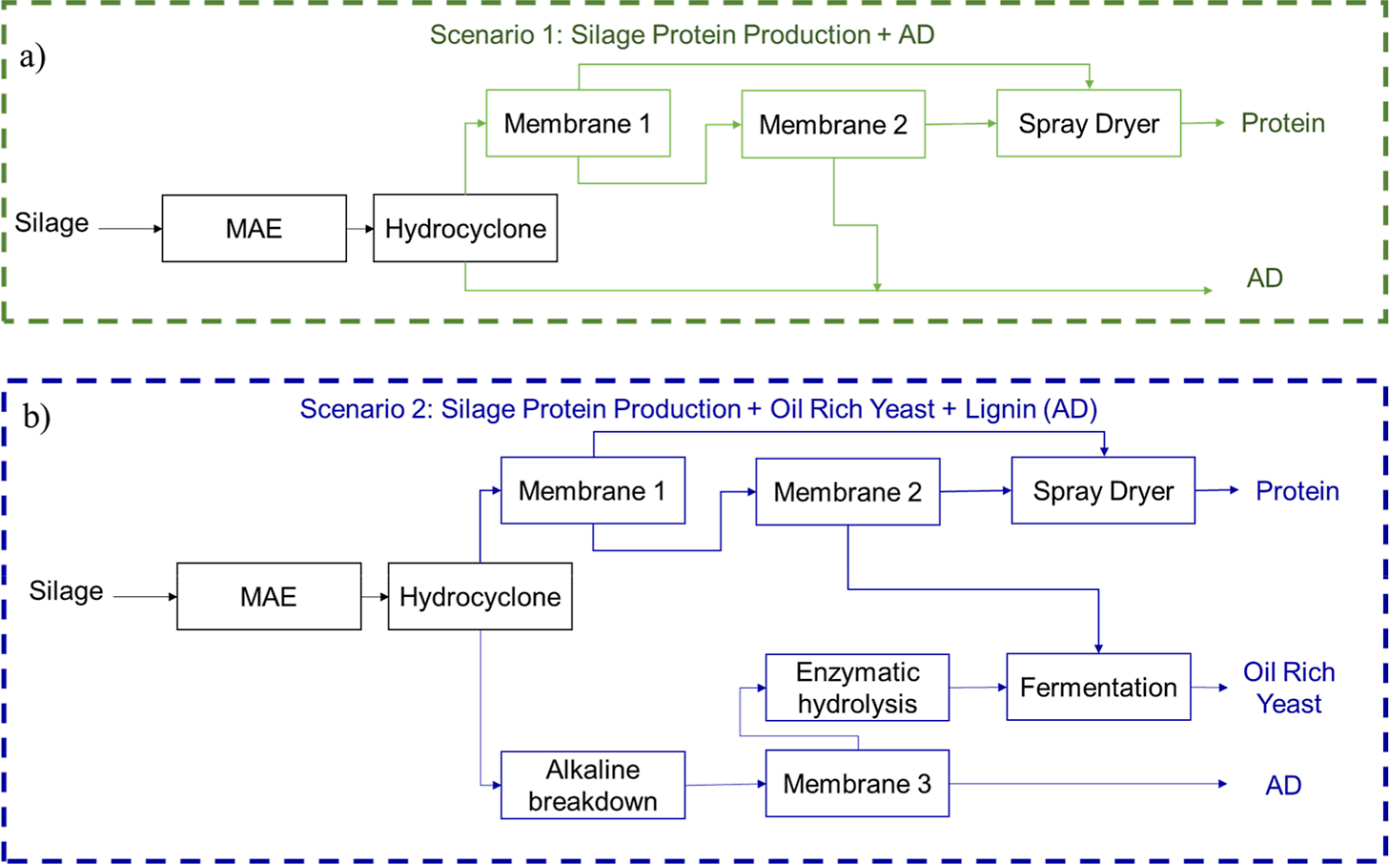
Process flowsheet for (a) Scenario 1: protein is extracted from silage via MAE and membrane filtration, with residual biomass sent to anaerobic digestion (AD), and (b) Scenario 2 introduces a fermentation step to convert hydrolyzed carbohydrates into microbial lipids prior to AD of the lignin-rich residue.

#### 2.2.2 Scenario 2—Protein + Lipid Co-Production via Fermentation + AD

This scenario builds upon the core protein extraction steps but implements a secondary valorisation route for the residual biomass (Figure 1b). Following protein extraction, the residual solid fraction, rich in cellulose and hemicellulose, is pretreated with dilute alkali and enzymatically hydrolyzed to release fermentable sugars. Experimental studies showed that the resulting hydrolysate supported robust growth of the oleaginous yeast *M. pulcherrima*, producing ∼10 g lipid/100 g silage.^11^ The reported lipid yield reflects experimentally observed system-level performance under optimized conditions and is therefore treated as an empirical input to the techno-economic model rather than a stoichiometric maximum. The yeast biomass contained over 40 wt % lipids, with a fatty acid profile comparable to high-oleic vegetable oils. Lignin-rich residues are routed to energy recovery pathways (e.g., AD via external facilities), without assuming high biodegradability or methane yields. AD remains the terminal step for lignin-rich residues, enabling energy recovery from otherwise recalcitrant biomass. This pathway complements the more advanced fermentation stage and enhances overall resource circularity by integrating both high-value and base-load energy products.

### 2.3 Techno-economic Assessment

The economic viability of both process scenarios was evaluated using a stochastic techno-economic modeling framework. Rather than relying on fixed-point estimates, key capital and operational cost parameters and output prices were modeled as uncorrelated probability distributions to reflect the early stage nature of the design. Monte Carlo simulations (*n* = 10,000) were used to propagate these uncertainties and estimate distributions for total capital investment (TCI), total production cost (TPC), and net present value (NPV).^12^ This enabled a risk-informed comparison between the baseline protein-only process (Scenario 1) and the integrated protein–lipid system (Scenario 2).

#### 2.3.1 Total Capital Investment

Equipment purchase-costs for a 5000 t protein/year biorefinery are detailed in Table S5. Total capital investment (TCI) was estimated following a factored cost approach. Base equipment purchase costs were derived from vendor quotes and prior plant design studies, then scaled using direct cost multipliers for piping, electrical, foundations, utilities, and other installation factors. The cost factors, adopted from Garrett (1989), are summarized in Table S6: TCI does not include anaerobic digestion infrastructure, under the assumption that solid residues are valorised externally.

#### 2.3.2 Total Operating Costs

TPC details are shown in Table S7, but they include:

- Raw materials (e.g., enzymes, sodium carbonate, sodium hydroxide, silage feedstock)
- Utilities (steam, water, and electricity)
- Labour, maintenance, and membrane replacement
- Fixed costs (overhead, insurance, etc.)

Raw material prices and annual usage were estimated from process flow rates and documented in Table S8. Price ranges reflect commercial variability and vendor quotations.

#### 2.3.3 Economic Analysis

The net present value (NPV) was calculated as:

1 $$NPV=\sum_{t=1}^{T}\frac{R_{t}-C_{T}}{{\left(1+r\right)}^{t}}-TCI$$

where *R*_*t*_ is the annual revenue, *C*_*t*_ is the annual costs, *r* = the discount rate and *T* the 25 year operational life of the plant.

As an additional metric, the unit production cost of protein was computed as:^13,14^

2 $$Unit\;\mathrm{cost}=\frac{Annualised\;TCI+TPC}{Annual\;protein\;output}$$

The techno-economic model represents an early stage process design intended to explore feasibility and key performance drivers rather than provide a fully bankable cost estimate. A plant lifetime of 25 years and a discount rate of 8% were assumed, with no salvage value assigned at end of life. Anaerobic digestion infrastructure is excluded from the capital cost on the basis that residual solids are valorised through external, third-party facilities. Key economic and process parameters were modeled as independent probability distributions. While correlations may exist in practice (e.g., between scale and energy demand or enzyme use and hydrolysis yield), robust correlation data are not available at this development stage. Assuming independence therefore avoids introducing speculative dependencies and is consistent with standard practice in early stage techno-economic assessments.

#### 2.3.4 Sensitivity and Scenario Analysis

One-at-a-time (OAT) sensitivity analyses were performed for (i) key parameters (input and output prices), (ii) protein extraction efficiency, and (iii) plant production scale (both scenarios). In each case, the resulting shifts in expected net present value (ENPV) and required break-even protein selling prices were analyzed. Exploratory runs were also conducted to assess the influence of varying the discount rate (6–12%) on financial performance, highlighting the critical role of access to low-cost capital for deployment.

#### 2.3.5 Feedstock Supply Simulation

To evaluate the logistical feasibility and delivery cost of silage, a spatial feedstock supply model was implemented. The model simulates radial sourcing of silage from harvestable land surrounding a biorefinery while excluding a 5 km nonharvestable zone (e.g., infrastructure, forests, urban areas). It assumes silage is transported from throughout the catchment, with transport distances adjusted using a tortuosity factor of 1.45 to reflect real-world road networks. The model allows for only a fraction of land within the catchment to be allocated to silage production (e.g., 30%), acknowledging the coexistence of other agricultural land uses. Monte Carlo simulations (*n* = 1000) were performed to evaluate uncertainty and variability in land use availability, biomass yield, and transport-related costs. Each iteration calculated an economically viable delivered silage cost (£/wet tonne at 25% dry matter), assuming return trips for all vehicle movements and allowing for the opportunity costs of switching to silage production. This supply side modeling supports the assumption of sourcing 33,333 t y^–1^ of silage from a decentralised agricultural landscape.

## 3 Results and Discussion

### 3.1 Total Capital Investment and Total Product Cost

Figure 2 shows the probability distributions for total capital investment (TCI) for both process scenarios. These probability distributions are based on normally distributed cost parameters with defined truncation limits to avoid nonphysical values; full parametrization and boundary conditions are provided in the Methodology. TCI refers to the upfront capital required to design, procure, and commission the facility, including process equipment, utilities, installation, and indirect costs such as contingency and engineering fees. Note that no salvage or terminal value is assumed for the biorefinery at the end of its 25 year operational life; assets are considered to have zero residual value, and decommissioning costs are excluded. This assumption reflects early stage uncertainty in end-of-life asset recovery and disposal costs. Moreover, the cost model excludes ancillary infrastructure essential to operation—such as feedstock and product storage, internal logistics, utilities support systems, and regulatory compliance facilities—which are expected to be site- and scale-dependent and therefore not explicitly costed at this early design stage.

**Figure 2:**
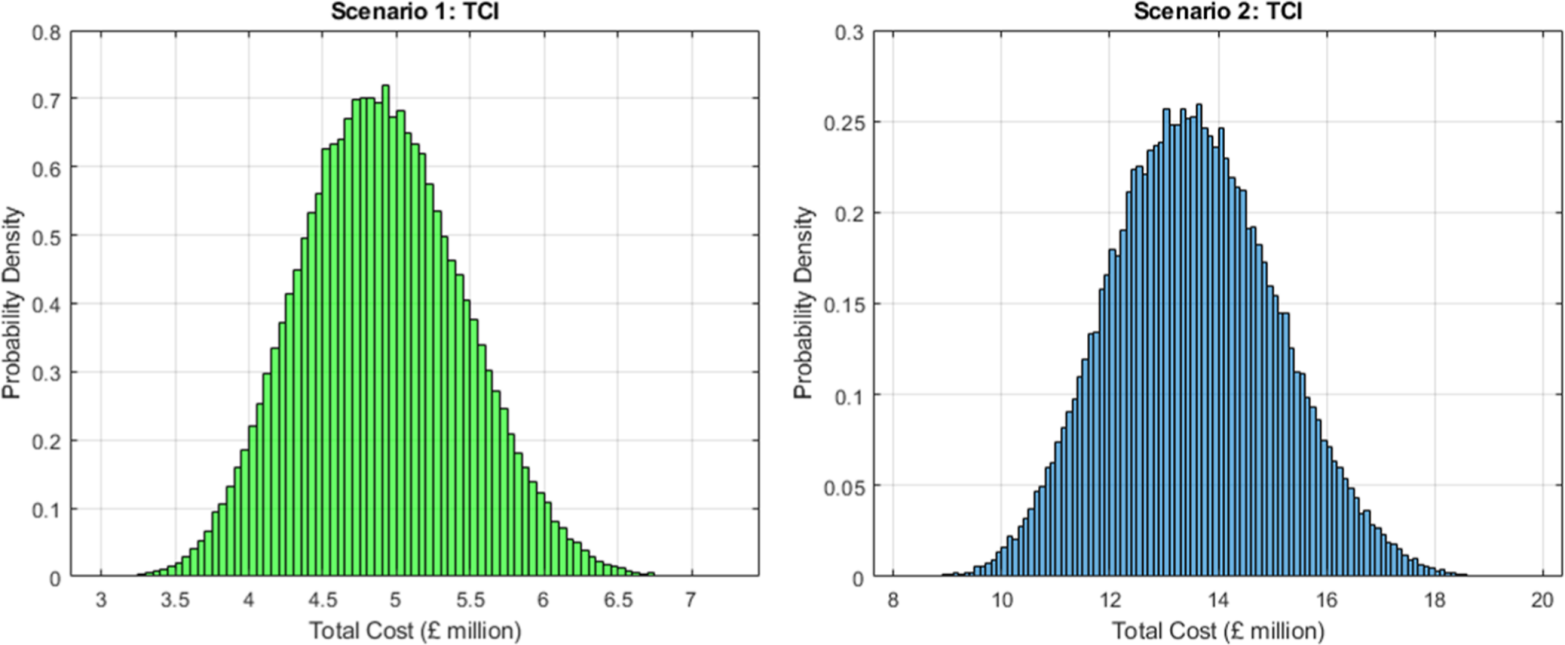
TCI distributions for Scenario 1 (left) and Scenario 2 (right). Median TCI is £4.8 million and £13 million, respectively. AD infrastructure is excluded. Scenario 2 shows greater variability due to added biological processing steps.

In Scenario 1, which focuses on protein production and off-site valorisation of the residual biomass, the median TCI is approximately £4.8 million, with a 90% confidence interval between £3 million and £5.8 million. The process configuration includes mechanical pretreatment, extrusion, solid–liquid separation, and membrane-based purification. AD is not included in the investment cost, as the residual solids are assumed to be sold or supplied to a third-party AD facility. This approach reduces capital intensity and aligns with decentralised, modular deployment models. In Scenario 2, which adds enzymatic hydrolysis, microbial fermentation, and lipid recovery, the median TCI rises to approximately £13.5 million, with a broader 90% confidence interval from £11 million to £16 million. For context, reported TCI for conventional legume-based protein facilities typically range from tens to hundreds of millions of pounds, reflecting more extensive purification, drying, and solvent-handling infrastructure. The lower TCI estimated here (£4.8–13.5 million) arises from the simplified process configuration, exclusion of anaerobic digestion infrastructure, and the production of a protein concentrate rather than a highly purified isolate.^14,15^ Direct comparisons should therefore be interpreted cautiously, given differences in scope and system boundaries.

The added cost reflects the inclusion of bioreactors, hydrolysis tanks, lipid separation systems, and expanded utilities, as well as higher contingency factors associated with biological process scale-up. The difference in capital exposure highlights a strategic design choice: Scenario 1 prioritises simplicity and external valorisation partnerships, while Scenario 2 internalizes more value creation at the cost of greater technical and financial risk. The broader TCI distribution in Scenario 2 further reflects early stage uncertainty in integrating multiple biological steps at commercial scale.

Figure 3 presents the probability distributions for total production cost (TPC), defined as the annual cost required to operate the facility and deliver the target protein output. TPC includes direct costs (raw materials, energy, labor, membrane replacement, fermentation media), indirect costs (maintenance, cleaning, utilities), and fixed charges (insurance, depreciation, and administrative overheads).

**Figure 3:**
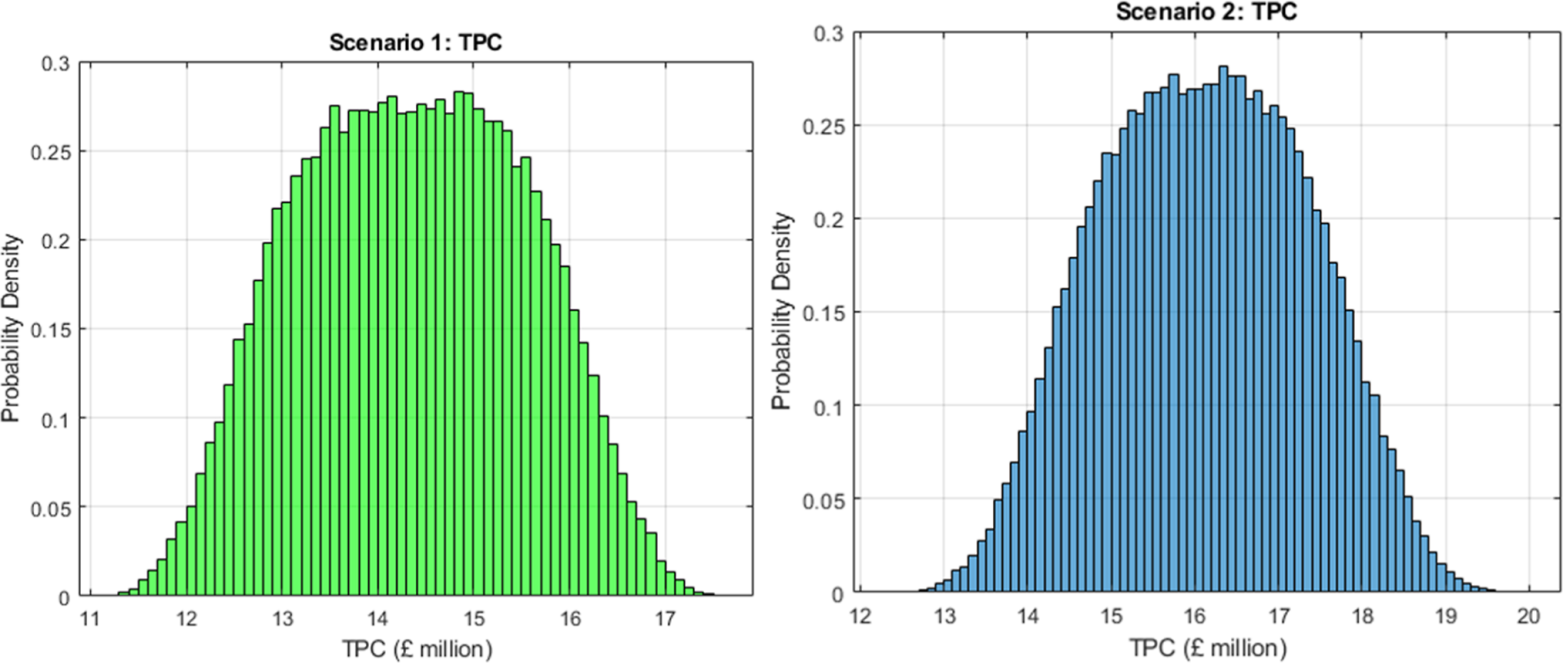
Total production cost (TPC) distributions for Scenario 1 (left) and Scenario 2 (right).

In Scenario 1, the median TPC is approximately £14 million/year, with a 90% confidence interval ranging from £11 million to £17 million/year. This relatively narrow distribution reflects the maturity of the core unit operations—mechanical pretreatment, twin-screw extrusion, and membrane filtration—and the exclusion of AD infrastructure from the cost basis. The system is primarily influenced by energy consumption (for drying and extrusion), membrane fouling rates, and throughput reliability. Membrane replacement and fouling control contribute significantly to operating costs, highlighting the importance of fouling-resistant materials and cleaning-in-place (CIP) strategies for long-term process optimization. Membrane replacement costs reflect assumed operational lifetimes and fouling rates typical of industrial filtration systems, with cleaning-in-place strategies implicitly represented through operating-cost variability. Fermentation-related risks, including contamination or batch failure, are not modeled as discrete events but are captured indirectly through increased uncertainty in operating costs and yields, consistent with early stage techno-economic assessments.

Scenario 2 exhibits a broader TPC distribution, with a median of £16 million/year and a 90% confidence interval between £12 million and £20 million/year. The elevated costs stem from the addition of enzymatic hydrolysis and microbial fermentation. Enzyme procurement, microbial nutrient supply (e.g., nitrogen, trace elements), temperature and pH control for fermentation, and downstream lipid recovery (e.g., centrifugation, solvent use or filtration) all contribute to the increased operational costs. Notably, the variance is higher than in Scenario 1, reflecting biological variability, sensitivity to nutrient pricing, and scale-up uncertainty typical of microbial bioprocessing. From a strategic standpoint, the TPC distribution informs both financial planning and risk management without considering full NPV values. For instance:

- Scenario 1 may be better suited to risk-averse investors or infrastructure-constrained regions, given its predictable cost structure and reduced dependency on bioreactor operation.
- Scenario 2, while more expensive, offers more diversification benefits and is more robust to market volatility in protein pricing, due to its multirevenue potential (protein, lipid and solids for AD).

Importantly, neither scenario includes the cost of anaerobic digestion (AD), which is assumed to be handled off-site through external partnerships. This assumption reduces the total cost exposure of the plant but also assumes a reliable market or contractual mechanism for selling or supplying lignocellulosic residue to AD operators. Any fluctuation in off-take price or regulatory compliance for digestate handling could alter TPC outcomes, particularly in Scenario 1. Finally, both TPC distributions will directly influence the minimum selling price (MSP) for the protein product and the overall net present value (NPV) under stochastic conditions, which is discussed in the following section.

Median TPC is £14 million/year for Scenario 1 and £16 million/year for Scenario 2. Scenario 2 reflects higher and more variable costs due to fermentation and lipid recovery.

### 3.2 Net Present Value and Risk Profile

Net present value (NPV) distributions were calculated for each scenario based on Monte Carlo simulations incorporating uncertainty in capital investment, operating costs, and market prices. The assessment was performed over the assumed 25 year operational life of the biorefinery using a discount rate of 8% with 2024 as the base year. Product prices were modeled as normal distributions to reflect both market variability and uncertainty in long-term pricing. For the protein powder, a mean price of £15/kg was assumed, with a standard deviation selected to produce a 95% confidence interval spanning £10/kg to £20/kg. The assumed protein price range reflects reported market values for plant-based protein concentrates such as pea, soy, and fava bean, particularly in food formulation and meat-alternative applications. These values are consistent with commercially available protein ingredients of comparable purity and functionality, while acknowledging ongoing price volatility in emerging alternative protein markets.^16,17^ In Scenario 2, microbial lipid-rich biomass was assigned a mean market value of £3/kg, with a truncated normal distribution varying between £2/kg and £5/kg. This reflects current pricing benchmarks for food-grade microbial oils and high-oleic alternatives to vegetable or algal oils, which are used in nutrition, feed, or specialty oleochemical markets. Finally, the lignocellulosic residues not valorised on-site were assumed to be sold to third-party AD operators. A selling value of £0.06–£0.10/kg of solid was applied to these solids, based on biogas energy equivalence and current gate-fee offsets observed in decentralised AD systems. While relatively low in monetary value, this stream helps avoid disposal costs and contributes to circular system design, particularly in Scenario 1 where coproduct revenue is otherwise limited. These price distributions were incorporated into the Monte Carlo simulation to capture the compounded impact of market variability on financial outcomes. Market volatility remains an important consideration, particularly for protein and microbial lipid products competing with established plant-protein supply chains. The stochastic pricing framework used here partially captures this uncertainty; however, sustained premium pricing would ultimately depend on product differentiation, functionality, and downstream market development. The present analysis therefore focuses on techno-economic feasibility rather than market forecasting. The resulting NPV distributions (Figure 4) reflect not only internal process economics but also external market sensitivity, particularly for protein pricing in Scenario 1 and lipid pricing in Scenario 2.

**Figure 4:**
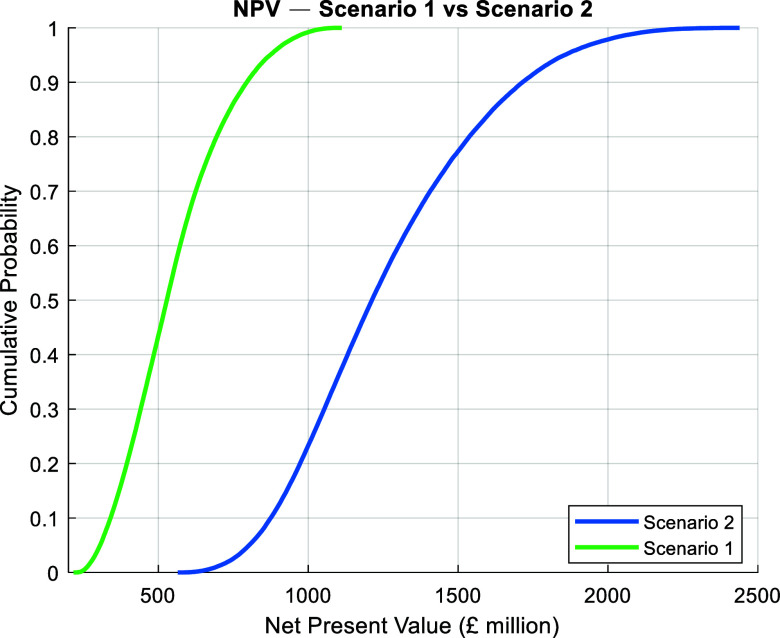
Cumulative probability distributions of net present value (NPV) for Scenario 1 (left, green) and Scenario 2 (right, blue).

All simulations produced positive NPV values for both scenarios, indicating strong underlying profitability across the entire range of economic assumptions. The median NPV for Scenario 1 was approximately £528 million, with a relatively narrow distribution, reflecting dual revenue stream from biomass AD and protein as well as the lower capital exposure. Scenario 2 achieved a median NPV of approximately £1,212 million, corresponding to roughly a 2.3-fold improvement in value over Scenario 1, driven by the additional revenue from microbial lipid production. However, this uplift requires a higher investment with nearly three times more upfront investment.

The cumulative NPV curves (Figure 4) illustrate that Scenario 1 delivers consistent returns with less capital at stake. Its narrower probability distribution appeals to investors seeking faster deployment, simpler operations. Scenario 2 introduces greater financial and technical complexity but offers significantly higher returns. Its broader NPV spread reflects both the variability of biological processes and the potential for an additional higher valued revenue stream. Whether Scenario 2 is the better investment ultimately depends on the decision-maker’s priorities. For capital-constrained or risk-averse investors, Scenario 1 may offer a faster path to commercialization with lower exposure. However, for investors with longer-term outlooks, greater capital flexibility, and access to lipid markets, Scenario 2 dominates in value creation potential, especially as fermentation technologies mature and microbial lipids gain wider adoption.

In addition to NPV-based profitability, the unit cost of protein production was calculated (see eq 2, Methodology). For Scenario 1 the estimated unit cost was approximately £2.97/kg of protein powder, whereas Scenario 2 incurred a higher cost of £3.40/kg, reflecting its greater capital intensity and process complexity. These values align with literature estimates for early stage protein platform production (e.g., ∼$2.99/kg in algal protein TEA^18^). Although Scenario 2 has the higher per-kg cost, it also delivers greater overall revenues thanks to the additional lipid-co-product and broader value streams. Thus, while Scenario 2 may suit higher-value markets where premium pricing is available, Scenario 1—with its lower production cost—may be better positioned for bulk or infrastructure-constrained regions where cost competitiveness is critical. Note that the higher unit protein cost in Scenario 2 reflects additional capital and operating costs associated with fermentation and lipid recovery, rather than differences in the protein extraction process itself.

### 3.3 Sensitivity Analysis and Performance Levers

A one-at-a-time (OAT) local sensitivity analysis was conducted to identify key input parameters driving economic outcomes. Each key input variable was varied across its defined uncertainty range while holding all others constant at their median values. The resulting change in expected net present value (ENPV)—the probability-weighted mean of all simulated NPV outcomes—was computed to assess the magnitude and direction of influence (Figure 5). For Scenario 1, the protein selling price had the most dominant effect on ENPV, with variations between its upper and lower bounds producing a total swing of nearly £450 million. This strong dependence reflects the central role of protein as the primary revenue stream in this configuration. The discount rate was the second most influential factor, where increasing it from 6% to 12% reduced ENPV by over £200 million, underscoring the significance of financing conditions and perceived investment risk. Other cost parameters—such as electricity, raw materials, silage price, and lignin price—had comparatively minor effects, each contributing less than £50 million variation in ENPV across their respective uncertainty ranges. Overall, Scenario 1 demonstrates relatively low sensitivity to operational inputs, consistent with its simpler configuration and limited number of revenue streams. Scenario 2 exhibits broader economic exposure due to its multiproduct structure. The oil-rich yeast price emerged as the most powerful lever, with ENPV shifts approaching ± £450 million across the evaluated range. The protein price and discount rate followed closely, with ENPV changes of roughly ± £300–400 million, confirming that both product pricing and financing costs dominate investment outcomes. Sensitivity to electricity and raw material costs was moderate, while silage and lignin prices contributed negligibly to the overall ENPV variance. The stronger dependence on market-driven variables indicates that Scenario 2, while more profitable on average, carries higher variability to commodity price volatility.

**Figure 5:**
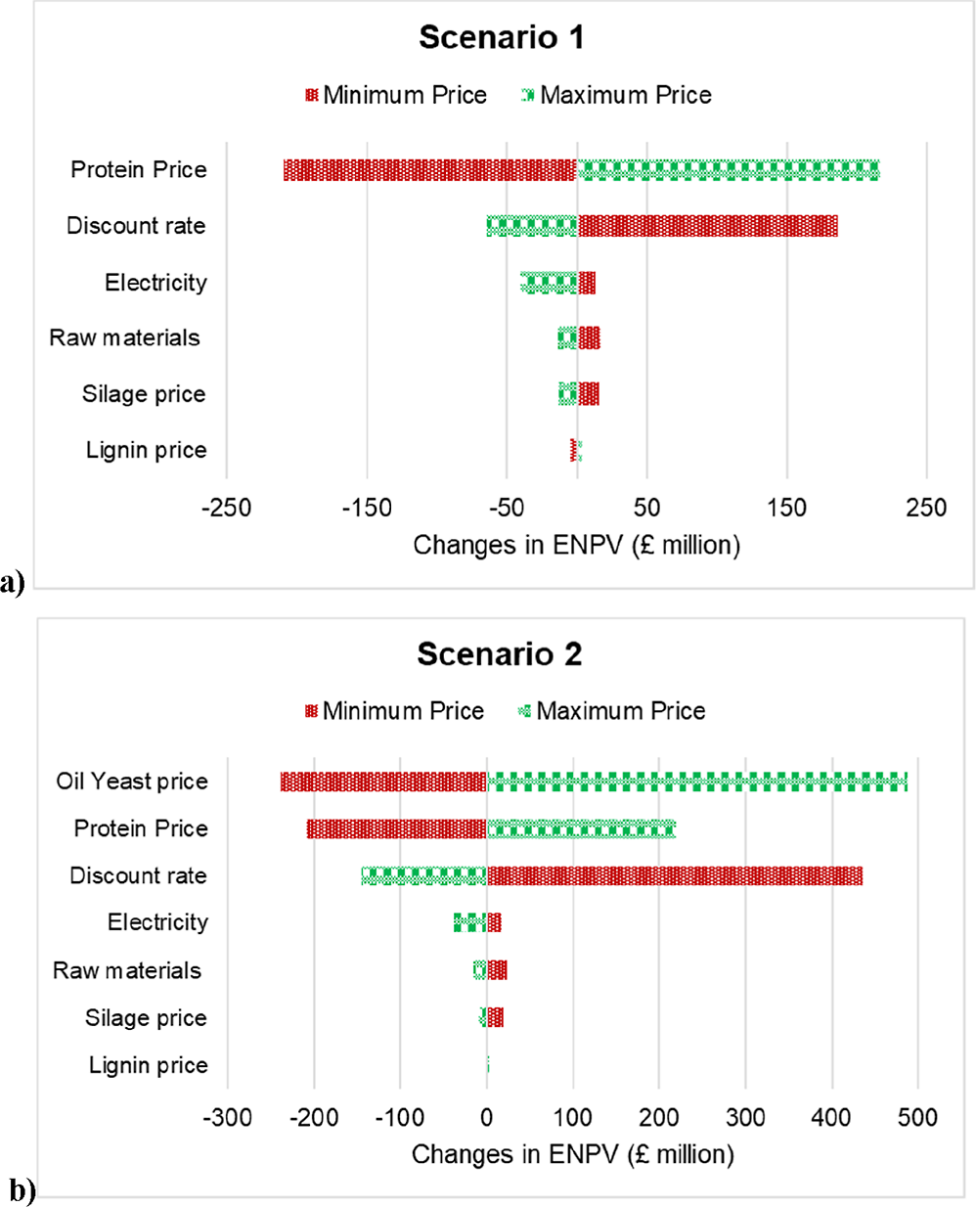
One-at-a-time sensitivity analysis for (a) Scenario 1 and (b) Scenario 2 showing the impact of each input variable on expected net present value (ENPV).

Taken together, these results highlight that Scenario 1 benefits from a stable but narrower revenue base, whereas Scenario 2 leverages multiple coproduct streams—protein and microbial oil—but at the expense of increased operational and market sensitivity. Improvements in process yields, product valorisation, or long-term offtake agreements for lipids could significantly reduce this volatility. Access to low-cost capital or public funding remains a key enabler for both configurations, as lowering the discount rate consistently improved ENPV. To better understand how the different scenarios respond to key design variables, one-at-a-time sensitivity analyses were performed for two technical levers: protein extraction efficiency and plant production scale. Both directly influence throughput, product yield, and process economics, offering clear guidance for scale-up and optimization strategies.

#### 3.3.1 Protein Extraction Efficiency

Extraction efficiency was evaluated as a major operational lever influencing overall project profitability, comparing multiproduct (protein + biomass for AD ± lipid coproduct) and protein-only revenue configurations (Figure S4). The results show distinct sensitivities and economic dynamics between the two process scenarios. In Scenario 1, increasing protein extraction from 0 to 15 g per 100 g silage raised the expected net present value (ENPV) from negative values to approximately £500 million. The multiproduct case, which includes biomass valorisation in anaerobic digestion, consistently yielded a modest premium—about £20–40 million—over the protein-only configuration at higher recoveries. This benefit demonstrates the stabilizing influence of coproduct utilization, even in the simpler process. The ENPV profile exhibits diminishing marginal returns beyond roughly 10 g protein per 100 g silage, suggesting that additional yield improvements yield progressively smaller financial gains. In Scenario 2, which integrates protein, biomass, and microbial oil revenue streams, the response to extraction efficiency was substantially stronger. ENPV increased from approximately £600 million at zero extraction to beyond £1.2 billion at 15 g protein per 100 g silage in the multiproduct configuration. When restricted to protein-only revenue, ENPV peaked at a much lower value, confirming that the full economic potential of Scenario 2 depends on reliable coproduct valorisation, particularly the lipid stream. The wide ENPV gap between multiproduct and protein-only cases at low extraction efficiencies highlights the risk-buffering role of diversified revenue pathways. These trends align with literature observations: high extraction yields are not just necessary for maximizing revenue but also for enabling more favorable process economics and lowering unit costs in protein systems (e.g., EAEP processes achieving 80% extractability delivered lower unit costs in bean protein models).^19^ Meanwhile, biorefinery studies emphasize that coproduct streams significantly improve financial resilience and reduce sensitivity to primary product pricing.^20^

To further quantify this relationship, the marginal economic benefit of extraction improvements was calculated (Table 2). Across both scenarios, a 1 g protein per 100 g silage increase in extraction yield produced an average ENPV gain of £40–50 million, with nearly linear growth between 3 and 12 g protein per 100 g silage and diminishing returns thereafter. While both systems exhibited similar marginal gains, Scenario 2 achieved higher absolute value due to the compounding effects of its lipid and biomass coproducts. Additional sensitivity results are provided in Figure S4 to support the interpretation of these results.

**Table 2:** Marginal Effect of Protein Extraction Efficiency on ENPV for Both Scenarios

protein extraction range (g protein/100 g silage)	ENPV change (Scenario 1, £ million)	marginal gain (Scenario 1, £ million/g)	ENPV change (Scenario 2, £ million)	marginal gain (Scenario 2, £ million/g)
0 → 3	+125	41.7	+120	40.0
3 → 6	+125	41.7	+150	50.0
6 → 10	+150	37.5	+150	37.5
10 → 12	+100	50.0	+100	50.0
12 → 15	+130	43.3	+110	36.7
Average	—	≈43 ± 5	—	≈43 ± 6

#### 3.3.2 Plant Scale

The effect of production scale on economic performance was assessed for plant capacities ranging from 1000 to 100,000 tonnes protein per year (Figure 6). Both configurations exhibited pronounced scale dependence, with increasing capacity leading to substantial gains in expected net present value (ENPV) and reductions in unit production cost. For Scenario 1, ENPV rose from near breakeven at 1000 t y^–1^ to approximately £10 billion at 100,000 t y^–1^, reflecting strong economies of scale in capital amortisation and process throughput. The associated unit protein price decreased modestly—from ∼£3.2/kg to below £2.9/kg—indicating that operating and capital efficiencies begin to plateau at higher capacities. In Scenario 2, which includes additional revenue from oil-rich yeast and biomass for AD coproducts, the scaling effect was even more pronounced. ENPV increased from below zero at 1,000 t y^–1^to nearly £20 billion at 100,000 t y^–1^, more than double that of Scenario 1. The unit price of protein decreased from roughly £3.9/kg to £3.1/kg, demonstrating how integrated multiproduct valorisation enhances profitability while buffering against fixed-cost dominance. These observations align with techno-economic studies of protein and bioproduct refineries, where scale-up beyond 10,000 t y^−1^ typically shifts capital cost contributions from >60% to <40% of total cost, and unit costs decline by 15–25% per order-of-magnitude increase in capacity.^21,22^ Similar patterns have been observed in algal and legume protein biorefineries, where integrated lipid or fiber valorisation improves investment resilience and reduces break-even thresholds.^23^

**Figure 6:**
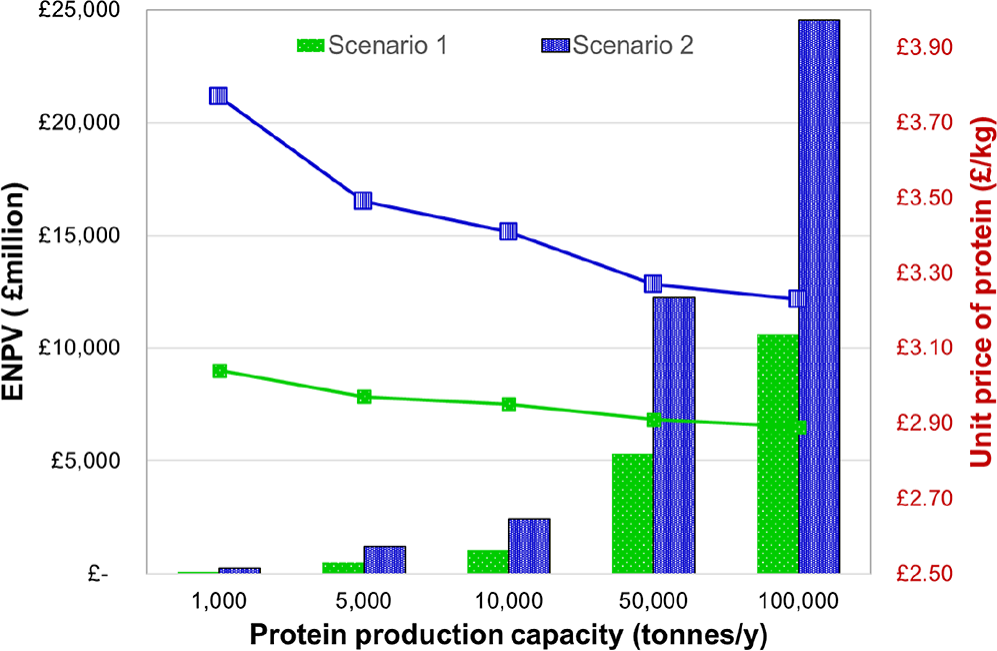
Effect of production capacity on expected net present value (ENPV) and break-even unit protein price for both Scenario 1 (green) and Scenario 2 (blue).

### 3.4 Sustainability Considerations

While a full life cycle assessment is beyond the scope of this study, the following discussion provides a qualitative perspective on sustainability implications based on process design choices and system integration. Both scenarios evaluated in this study offer substantial environmental advantages over conventional protein production pathways, particularly those based on animal agriculture, while differing in their resource efficiency, energy integration, and circularity potential. Grass-based protein extraction leverages an underutilized biomass resource for human consumption. In the UK alone, more than 12 million hectares of grassland are maintained, with an estimated 20 million tonnes of harvestable biomass annually from non-upland terrain.^24^ Unlike increasing soy or pea production, switching to grass-based protein production would offer an opportunity for arable systems to introduce pasture leys for improving soil health or enable existing pasture-based systems to diversify. Scenario 1, centered on protein recovery, already achieves significant conversion efficiency. With a target recovery of 15 g protein/100 g silage, and minimal chemical inputs (e.g., sodium carbonate), the process transforms grass into a human-edible product while maintaining low freshwater and land footprints. Scenario 2 builds upon this by capturing additional value from structural carbohydrates via fermentation, resulting in higher total biomass utilization.

Both scenarios adopt circular design principles by routing remaining lignocellulosic residues to external anaerobic digestion (AD). This avoids landfill or low-value disposal and supports regional energy generation through biogas. In Scenario 2, the inclusion of microbial lipid coproduction enhances the circular profile by valorising sugars liberated during enzymatic hydrolysis, a key example of cascade use of biomass. The resulting system generates multiple product streams from a single feedstock, including protein, lipids, energy (biogas), and nutrient-rich digestate. This modular, flexible use of biomass aligns with EU and UK priorities for circular bioeconomy strategies.

In addition to evaluating process economics and sustainability, we assessed the logistical feasibility of securing silage feedstock at scale using a spatial supply model. The model assumes radial sourcing from surrounding grassland, excluding a 5 km nonharvestable buffer and applying a 1.45 tortuosity factor to account for real-world road access. Monte Carlo simulations incorporating regional land-use productivity indicate that sourcing 33,333 t y^–1^ of wet silage (25% dry matter) is achievable within modest transport distances and at economically viable prices. For regions dominated by livestock systems (e.g., Wales), the model yields an average delivered cost of £51.47 ± 19.67 t^–1^ (25% DM), equivalent to approximately £195.6 ± 74.8 t^–1^ on a 95% DM basis. In contrast, for arable regions (e.g., East of England), the corresponding delivered costs are £58.33 ± 22.66 t^–1^ (25% DM) or £221.0 ± 91.0 t^–1^ (95% DM). These equivalence prices assume constant profitability relative to baseline livestock or arable systems and therefore represent realistic opportunity-cost thresholds for growers. In general, these values align closely with the silage input-cost range used in our techno-economic model (£0.10–0.18 kg^–1^ wet basis), reinforcing the credibility of the deployment scale. Importantly, the spatial analysis suggests that a medium-scale biorefinery of the size evaluated here could be integrated into existing pasture-dominated landscapes without requiring major land-use change or new centralized infrastructure, provided regional biomass yields and logistics are appropriately managed.

While a full life cycle assessment (LCA) is beyond the scope of this study, preliminary estimates suggest that both processes have significantly lower greenhouse gas (GHG) intensity than animal protein systems. The exclusion of methane-emitting ruminants, reduced transport needs, and integration with local AD networks reduce both direct and indirect emissions. Energy demand remains a key consideration. Scenario 1 is dominated by thermal and mechanical loads from extrusion and drying, whereas Scenario 2 adds fermentation energy input and temperature control, partially offset by the potential to cogenerate heat or power from AD-derived biogas. Future work could explore energy symbiosis or heat recovery integration to further improve net energy balance. Future research should also employ a thorough cradle-to-grate life cycle assessment (LCA) in compliance with ISO 14040/44 standards to measure these benefits. This would assess energy consumption, eutrophication potential, land occupation, greenhouse gas emissions, and freshwater demand at all significant stages, including feedstock production, transportation, processing, product distribution, and end-of-life handling. Further, to benchmark the sustainability standards of grass-derived protein, a comparative LCA should incorporate soy, dairy and animal protein system scenarios.

Decentralised roll-out of grass-based protein systems offers an opportunity to relocalize UK protein production, reduce dependency on imports, and create additional value from regional biomass streams. Scenario 1 lends itself to modular deployment in rural or peri-urban settings, while Scenario 2 may be best suited for integration into existing industrial bioprocessing infrastructure. Both pathways contribute to diversifying the protein supply and lowering the environmental cost per unit of nutrition. Scenario 2, in particular, supports coproduct strategies that integrate food, feed, and energy objectives, positioning it as a cornerstone of a more circular, resilient bioeconomy.

## 4 Conclusions

This work demonstrates that grass-based biorefineries can convert an underutilized biomass into valuable protein and lipid products, providing a feasible route toward a regional circular bioeconomy. Three main lessons emerge: (1) process simplicity and modularity are as important as yield. While multiproduct integration (Scenario 2) offers higher returns, it also increases financial exposure; smaller modular systems (Scenario 1) may offer faster learning and easier replication during early deployment. (2) protein extraction efficiency is the dominant technical lever, with each additional gram of protein per 100 g silage increasing ENPV by about £40–50 million. Research should therefore focus on yield–cost coupling and process intensification that improve recovery without raising energy or enzyme demand. (3) Feedstock localization underpins scalability. Spatial modeling confirms that 33,333 t y^–1^ of silage can be sourced within short transport radii and at regionally competitive costs, supporting decentralised deployment without major land-use change.

Looking ahead, efforts should prioritise (i) improving extraction efficiency, (ii) stabilizing coproduct markets for microbial oils and digestate fertilizers, and (iii) integrating life-cycle and techno-economic modeling. Together, these strategies can accelerate the transition from feasibility to implementation of grass-based protein biorefineries within sustainable agricultural systems.

## Supplementary Material



## Notes

The Supporting Information provides additional methodological detail and data underpinning the techno-economic analysis, including feedstock composition and basis assumptions, process yield and mass-balance calculations for both scenarios, capital and operating cost breakdowns, parameter ranges used in the stochastic modeling, and supplementary sensitivity analyses. Detailed tables and figures are included to support transparency and reproducibility of the modeling framework without overloading the main manuscript (PDF)
