# Supplementary material for: From Grass to Protein: Assessing the Economic Viability of Mechanochemical-Assisted Extraction for Sustainable Food Production
Source: ACS Sustain Chem Eng. 2026 Jan 23;14(5):2676–86. doi: 10.1021/acssuschemeng.5c12483 (PMC12892315; doi:10.1021/acssuschemeng.5c12483)
Supplement: Supplementary file 1 [file sc5c12483_si_001.pdf]

Supplementary Information

Title of Manuscript:

From Grass to Protein: Assessing the Economic Viability of Mechanochemical-Assisted Extraction for Sustainable Food Production

Authors:

Bernardo Castro-Dominguez[a], Oscar Selway Mindrina[a], Madhurima Dutta[a], Yubin Ding[a], Karl Behrendt[b], Anne Wambui Mumbi[b,c] , Richard Green[c], Hannah S. Leese[a], Christopher J. Chuck[a]

Affiliations:

[a] Department of Chemical Engineering, University of Bath, Bath, BA2 7AY, UK.

[b] Harper Adams Business School, Harper Adams University, Newport, TF10 8NB, UK

[c] Department of Engineering, Harper Adams University, Newport, TF10 8NB, UK

Corresponding Author:

Dr Bernardo Castro Dominguez

bcd28@bath.ac.uk, Phone: +44 1223 38 4946

Description of Supporting Information:

Additional methodological details, compositional data, mass-balance calculations, and supplementary analyses supporting the techno-economic assessment presented in the main manuscript.

Contents Summary:

- **Total number of pages:** 12
- **Number of figures:** 4
- **Number of tables:** 8

Section SI-1 - Detailed nutritional and functional data

It is important to distinguish between the biomass basis used for experimental characterisation and that used for techno-economic modelling. Compositional analysis was performed on oven-dried silage (95% dry matter) to enable accurate biochemical characterisation. In contrast, all techno-economic calculations assume wet silage at 25% dry matter, consistent with realistic on-farm ensiling practices and feedstock logistics.

Table S1 Experimental silage extraction characteristics

Parameter	Silage	Extractive free silage
DM[1]	95% ±0.0%	92% ±0.0%
Total extractives%	42.54±2	100-42.54=**57.46**
Total Ash%	9.74±0.1	1.17 g
Total Lignin	-	7.77 g
Acid insoluble lignin (Klason)	-	6.01 g
Total Cellulose	-	25.26 g
Total Hemicellulose	-	11.53 g
Total Protein%	11.45±0.15	5.07 g
**Crude oil/fat*%**	2.38±0.13	
	Sum	**57.51**

[1]. Compositional values reflect dried silage samples analysed under laboratory conditions (95% dry matter), which differ from typical on-farm silage moisture levels. The protein content reported here (11.45 wt.%) represents this specific batch. For process modelling, a theoretical maximum of 15 wt.% protein was assumed to estimate best-case recovery potential and is later varied in sensitivity analysis.

*Crude fat analysis has been conducted on raw silage. Therefore, it is not included to the mass calculation

Table S2. Nutritional composition and functional properties of grass-derived protein concentrate obtained via mechanochemical-assisted extraction (MAE). [1]

Parameter	Unit	Measured value (mean ± SD)	Reference or comparison
**Protein recovery**	% of total protein in silage	52 ± 3 %	Comparable to soy isolate recovery in alkaline extraction (50–55 %)
**Protein purity (dry basis)**	wt %	60 ± 2 %	Target value used in TEA
**Essential amino acid profile**	g AA per 100 g protein	Histidine 2.4	Leucine 7.8
**Vitamins (water-soluble)**	mg per 100 g protein	B1 (Thiamine) 0.32	B2 0.28
**Functional properties**	–	Emulsifying capacity ≈ 70 m^2/g protein	Foaming capacity ≈ 35 %
**Ash**	wt % (dry basis)	8.5 ± 0.4 %	–
**Residual carbohydrate**	wt % (dry basis)	22 ± 3 %	–

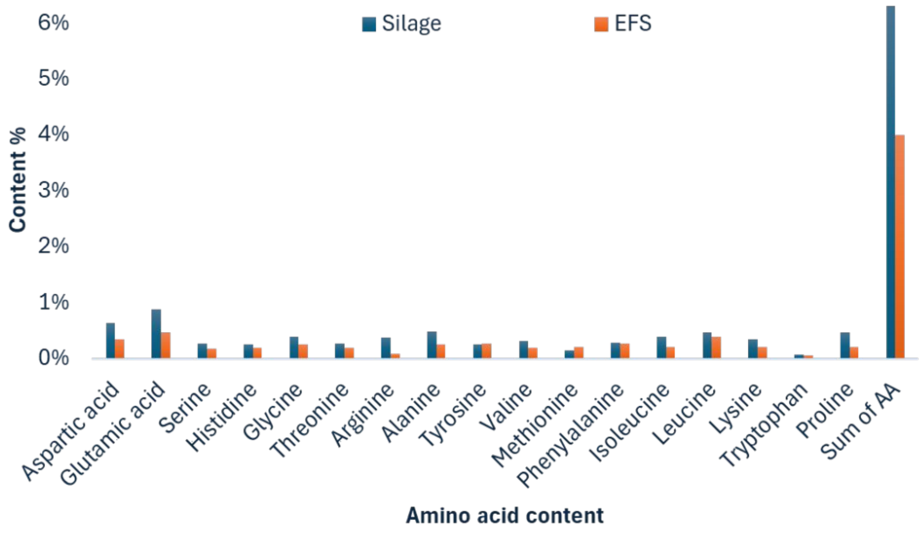

Figure S1 Experimental silage and extracted silage amino acid content

Section S2 – Process design and Mass and Energy Balances

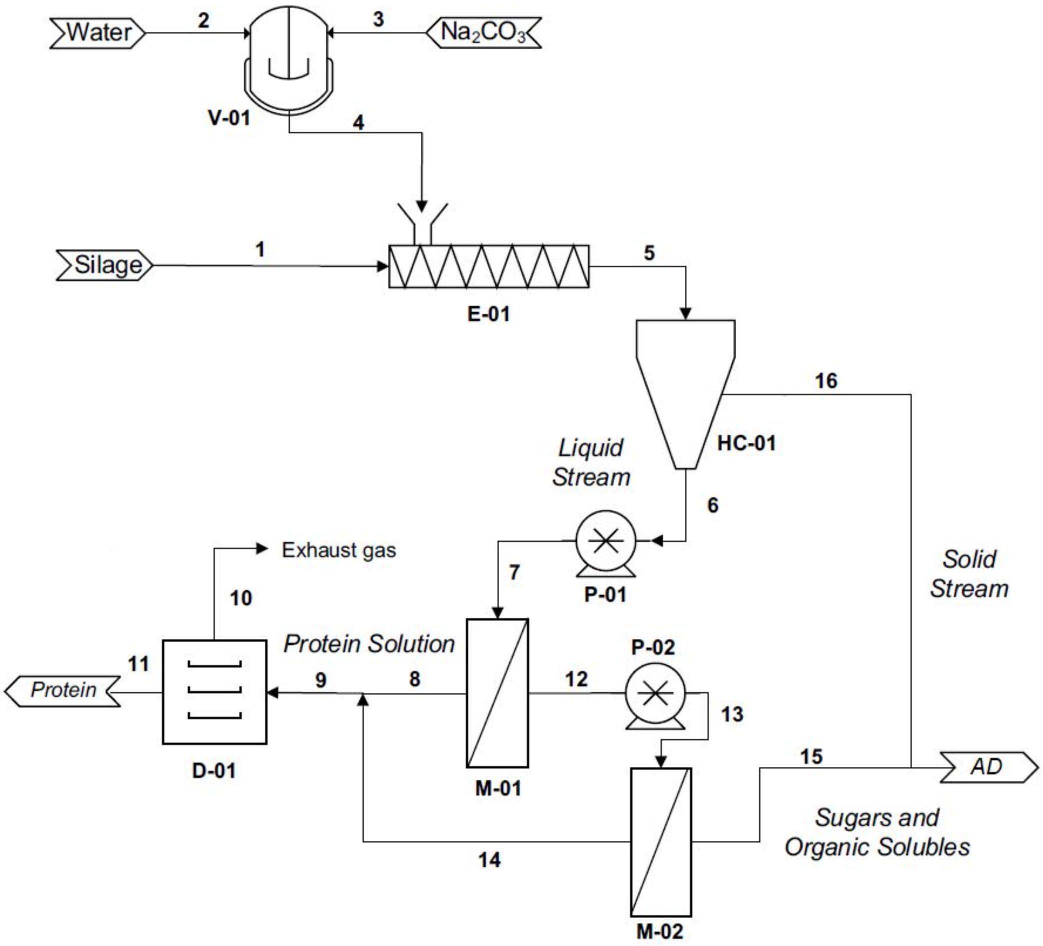

Figure S2 Process Flow diagram for Scenario #1

Table S3 Mass Balances Scenario #1

	**Mass Flow rate (kg/h)**																
**Stream**	**1***	**2**	**3**	**4**	**5**	**6**	**7**	**8**	**9**	**10**	**Protei n**	**12**	**13**	**14**	**15**	**16**	**AD***
Dry Silage*	3614	0	0	3614	0	0	0	0	0	0	0	0	0	0	0	0	0
Water	190	1359	0	1549	1549	1394	1394	558	725	725	0	837	837	167	669	155	824
Protein	0	0	0	0	542	488	488	244	343	0	343	244	244	99	145	54	199
Lignocellulose	0	0	0	0	485	4	4	4	4	0	4	0	0	0	0	481	481
Hemicellulose/Cellulose	0	0	0	0	2149	215	215	107	183	0	183	107	107	75	32	1934	1966
Sodium Carbonate	0	0	272	272	272	27	27	5	12	0	12	22	22	6	16	245	260
Ash	0	0	0	0	352	35	35	18	25	0	25	18	18	8	10	317	327
Other (Fat, Vitamins)	0	0	0	0	86	9	9	0	5	0	5	9	9	5	3	77	81
**Totals**	**3804**	**1359**	**272**	**5435**	**5435**	**2172**	**2172**	**936**	**1296**	**725**	**571**	**1236**	**1236**	**360**	**876**	**3263**	**4139**

[i] Dry Silage is assumed to be 95% DM
[ii] Water is added to attain a 30 wt.% moisture before mechanochemical treatment
[iii] Sodium Carbonate is added to attain a 5 wt.%
[iv] 60% purity of protein in the stream
*Dry biomass for AD = 4,139-824 = 3315 kg/h or 29,049 tonnes per year

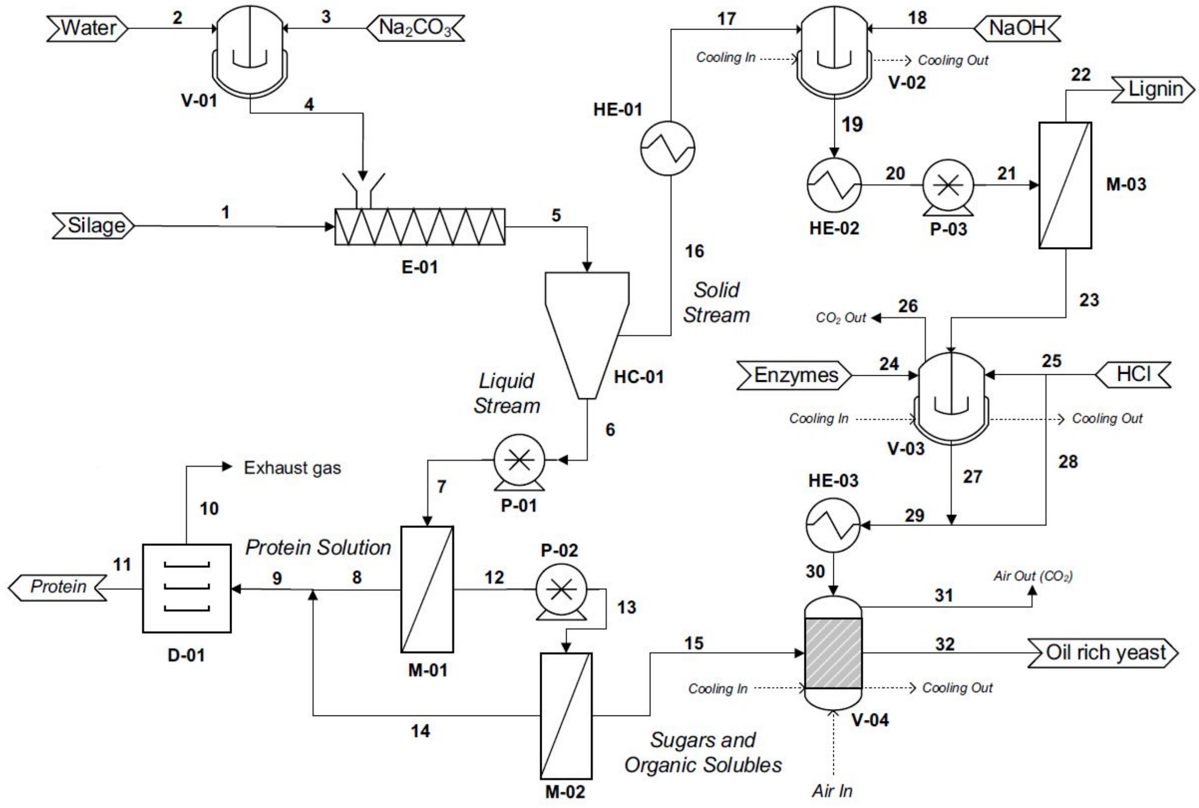

Figure S3 Process Flow diagram for Scenario #2

Table S4 Mass Balances Scenario #2

	Mass Flow rate (kg/h)															
**Stream**	**1**	**2**	**3**	**4**	**5**	**6**	**7**	**8**	**9**	**10**	**11**	**12**	**13**	**14**	**15**	**16**
Dry Silage*	3614	0	0	3614	0	0	0	0	0	0	0	0	0	0	0	0
Water	190	1359	0	1549	1549	1394	1394	558	725	725	0	837	837	167	669	155
Protein	0	0	0	0	542	488	488	244	343	0	343	244	244	99	145	54
Lignocellulose	0	0	0	0	485	4	4	4	4	0	4	0	0	0	0	481
Hemicellulose	0	0	0	0	2149	215	215	107	183	0	183	107	107	75	32	1934
Lignin	0	0	0	0	0	0	0	0	0	0	0	0	0	0	0	0
Cellulose	0	0	0	0	0	0	0	0	0	0	0	0	0	0	0	0
Glucose	0	0	0	0	0	0	0	0	0	0	0	0	0	0	0	0
Sodium Carbonate	0	0	272	272	272	27	27	5	12	0	12	22	22	6	16	245
Ash	0	0	0	0	352	35	35	18	25	0	25	18	18	8	10	317
Other (Fat, Vitamins, sugars)	0	0	0	0	86	9	9	0	5	0	5	9	9	5	3	77
Sodium Hydroxide	0	0	0	0	0	0	0	0	0	0	0	0	0	0	0	0
Enzymes	0	0	0	0	0	0	0	0	0	0	0	0	0	0	0	0
HCl	0	0	0	0	0	0	0	0	0	0	0	0	0	0	0	0
CO2	0	0	0	0	0	0	0	0	0	0	0	0	0	0	0	0
Lipid Rich Yeast Cells	0	0	0	0	0	0	0	0	0	0	0	0	0	0	0	0
**Totals**	**3804**	**1359**	**272**	**5435**	**5435**	**2172**	**2172**	**936**	**1296**	**725**	**571**	**1236**	**1236**	**360**	**876**	**3263**

	Mass Flow rate (kg/h)																
**Stream**	**17**	**18**	**19**	**20**	**21**	**Lignin**	**23**	**24**	**25**	**26**	**27**	**28**	**29**	**30**	**31**	**32**	**Oil Rich Yeast**
Dry Silage*	0	0	0	0	0	0	0	0	0	0	0	0	0	0	0	0	0
Water	155	0	155	155	155	3	152	0	0	0	152	0	152	152	0	821	0
Protein	54.2	0	54	54	54	0	54	0	0	0	54.2	0	54.2	54.2	0	199	199
Lignocellulose	481	0	0	0	0	0	0	0	0	0	0	0	0	0	0	0.0	0
Hemicellulose	1934	0	387	387	387	8	379	0	0	0	379	0	379	379	0	411	411
Lignin	0	0	476	476	476	386	91	0	0	0	91	0	90.5	90.5	0	91	91
Cellulose	0	0	1552	1552	1552	0	1552	0	0	0	155	0	155	155	0	155	155
Glucose	0	0	0	0	0	0	0	0	0	0	1397	0	1397	1397	0	458	1397
Sodium Carbonate	245	0	245	245	245	5	240	0	0	0	240	0	240	240	0	255	255
Ash	317	0	317	317	317	32	285	0	0	0	285	0	285	285	0	295	295
Other (Fat, Vitamins, sugars)	77	0	77	77	77	23	54	0	0	0	54	0	54.2	54.2	0	57	57
Sodium Hydroxide	0	0.62	0.62	0.62	0.62	0.06	0.56	0	0	0	0.56	0	0.56	0.56	0	0.6	1
Enzymes	0	0	0	0	0	0	0	14.9	0	0	14.9	0	14.9	14.9	0	15	15
HCl	0	0	0	0	0	0	0	0	0.01	0	0.01	0	0.01	0.01	0	0.0	0
CO2	0	0	0	0	0	0	0	0	0	0	0	0	0	0	559	0.0	0
Lipid Rich Yeast Cells	0	0	0	0	0	0	0	0	0	0	0	0	0	0	0	381	381
**Totals**	**3263**	**0.62**	**3264**	**3264**	**3264**	**456.56**	**2807**	**14.9**	**0.01**	**0**	**2822**	**0**	**2822**	**2822**	**559**	**3139**	**3257**

*3257 kg/h is ~28,541 tonnes/year

Section S3 – Economic Assessment

Table S5 Equipment listing with sizing and costing for a plant capable of producing 5000t/year of protein (or 33,333 t/year of silage)

Unit	Equipment	Costing strategy	No. Units	Total Purchase Costs (£)*
E-01	Extruders	1	1	612,900
HC-01	Hydro cyclone	2	1	1,400
M-01	Membrane 1	1	1	25,500
M-02	Membrane 2	1	1	24,000
M-03	Membrane 3	1	1	17,000
HE-01-3	Heat Exchangers	2	1	18,000
V-01	Vessel salt mix	3	1	23,000
V-02	Vessel alkaline	3	2	53,000
V-03	Vessel hydrolysis	3	12	550,000
V=04	Fermenter	3	9	615,000
D-01	Spray Dryer	2	1	94,000
			**Total Scenario 1**	**~750,000**
			**Total Scenario 2**	**~2,100,000**

1. Product and Process Design Principles' (Warren et al., 2017).
2.'A Guide to Capital Cost Estimating' (Gerrard, 1988).
3. From tender
*The most recent cost index was taken to be 789.2 from Maxwell (2020).
Note: Costs associated with feedstock storage and handling were not included explicitly in the equipment, as these are embedded in the intrinsic price assumed for the delivered silage feedstock.

Table S6: Direct costs from Garrett, D.E. (1989). *Chemical Engineering Economics*.[2]

Component	Plant cost factor
Purchased equipment	1.00
Piping	0.15 - 0.70
Electrical	0.10 - 0.15
Instrumentation	0.10 - 0.35
Utilities	0.30 - 0.75
Foundations	0.07 - 0.12
Insulation	0.02 - 0.08
Painting, fireproofing, safety	0.02 - 0.10
Yard improvements	0.05 - 0.15
Environmental	0.10 - 0.30
Buildings	0.05 - 1.00
Land	0.03 - 0.10

Table S7: Cost of raw materials. Price ranges reflect commercial variability and vendor quotations.

Raw materials	Mass flowrate (kg/year)*	Unit prices (£/kg)**
Enzymes	118,000	0.20 – 6.27
Hydrochloric acid	55	0.03 – 0.13
Silage (95% DM)	33,333,333	0.1 – 0.18
Sodium carbonate	4,900	0.19 – 0.33
Sodium hydroxide	800	0.20 – 0.48
Water	10,800,000	0.001 – 0.003

*All values were estimated from mass balance and experimental requirements
**All values were expressed in 2024 GBP.

Table S8. Breakdown of Total Production Cost (TPC) by major cost category for Scenario 1 and Scenario 2

Cost category	Description	Representative components	Scenario 1 (£ million y^{-1})	Scenario 2 (£ million y^{-1})
**Raw materials**	Consumables and process chemicals required for feed preparation and reaction	Silage feedstock, enzymes, Na_2CO_3, NaOH, microbial nutrients	5.4-6.5	5.5 – 7.0
**Utilities**	Energy and service inputs for unit operations	Process water, cooling water, electricity	3.1-5.4	3.3 – 6.0
**Labour**	Direct operating labour	Operators, supervisors	0.8-1.3<br><br>(28 workers)	1.1-1.5<br><br>(34 workers)
**Fixed costs & overheads**	Annualised indirect expenses	Administrative overhead, insurance, taxes, environmental compliance	3.2-4.1	4.8-5.7
**Total production cost (TPC)**	—	—	**11-18**	**13-20**

*Labour was estimated as N = (6.29 + 31.7 P^2 +0.23 N)^0.5; Where P and N are process steps with solids

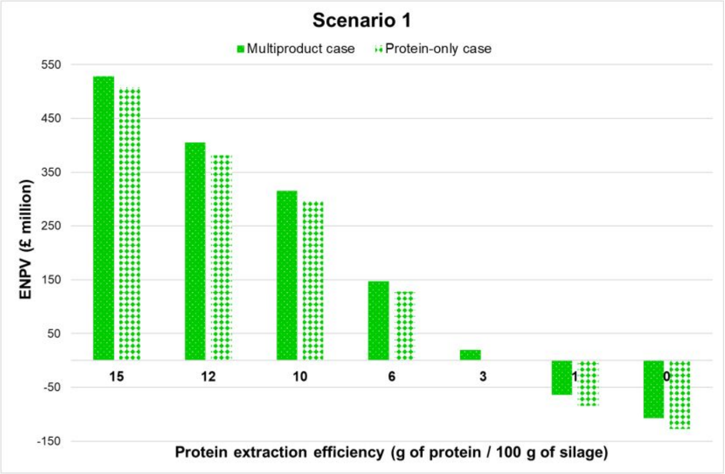

(a)

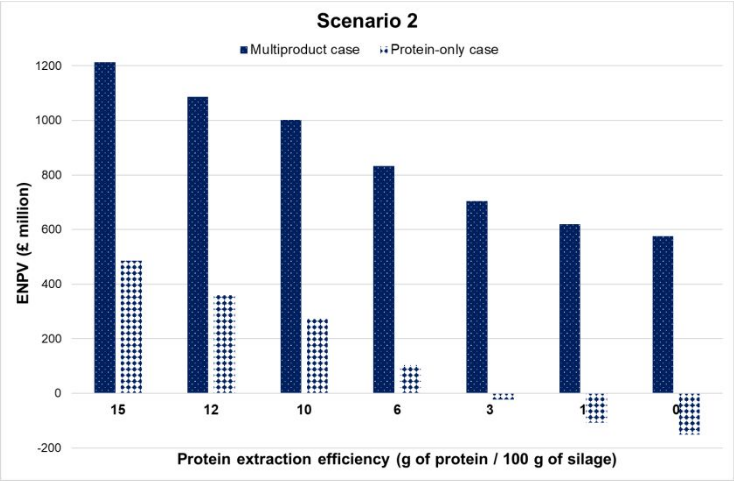

(b)

Figure S4. Influence of protein extraction efficiency on expected net present value (ENPV) for (a) Scenario 1 and (b) Scenario 2, under multiproduct and protein-only revenue cases.

[1] Olusegun Abayomi Olalere, Fatma Guler, Christopher J. Chuck, Hannah S. Leese, Bernardo Castro-Dominguez, Mechanochemical extraction of edible proteins from moor grass, RSC Mechanochemistry, Issue 4, 2024. https://doi.org/10.1039/D4MR00016A

[2] D. E. Garrett, Chemical Engineering Economics, Springer Netherlands 2012
